# Supplementary material for: Association between Osteoporosis and Cognitive Impairment during the Acute and Recovery Phases of Ischemic Stroke
Source: Medicina (Kaunas). 2020 Jun 23;56(6):307. doi: 10.3390/medicina56060307 (PMC7353884; doi:10.3390/medicina56060307)
Supplement: Supplementary file 1 [file medicina-56-00307-s001.pdf]

SUPPLEMENTARY DATA

Table S1. Baseline characteristics of the osteoporosis group and the no-osteoporosis group (using database 3, $n = 302$).

	Non osteoporosis ($n = 216$)	Osteoporosis ($n = 86$)	p-value
Age, years (SD)	71.7 (10.8)	77.3 (8.4)	0.05 †
Female (%)	101 (53.2)	76 (88.4)	<0.001 *
BMI, kg/m^2 (SD)	24.3 (3.9)	23.0 (3.4)	0.99 †
Initial NIHSS score (%)			0.93 *
0-4	134 (62.0)	52 (60.5)	
5~11	58 (26.9)	23 (26.7)	
>11	24 (11.1)	11 (12.8)	
Stroke subtype (%)			0.52*
SVO	50 (23.1)	24 (27.9)	
LAA	83 (38.4)	27 (31.4)	
CE	40 (18.5)	20 (23.3)	
Other	43 (19.9)	15 (17.4)	
Prior stroke (%)	53 (24.5)	25 (29.1)	0.47 *
Hypertension (%)	137 (63.4)	60 (69.8)	0.35 *
Diabetes mellitus (%)	83 (38.4)	25 (29.1)	0.14 *
Hyperlipidaemia (%)	33 (15.3)	12 (14.0)	0.86 *

Current smoking (%)	37 (17.1)	2 (2.3)	<0.001 *
Atrial fibrillation (%)	47 (21.8)	20 (23.3)	0.88 *
Prior antithrombotic agent use (%)	88 (40.7)	36 (41.9)	0.90 *
Education, years (SD)	7.1 (4.3)	6.7 (4.2)	0.19 †
mRS>2 at discharge (%)	63 (29.2)	28 (32.6)	0.58 *
mRS>2 at 3 months (%)	80 (37.0)	38 (44.2)	0.30 *
WBC, ×1000/dL (SD)	7.55 (2.51)	7.52 (2.53)	0.73 †
LDL, mg/dL (SD)	97.3 (34.5)	91.5 (28.1)	0.08 †
Haemoglobin, g/dL (SD)	13.8 (1.8)	13.5 (1.8)	0.75 †
Creatinine, mg/dL (SD)	0.97 (0.36)	1.06 (0.80)	0.10 †
HbA1c, % (SD)	6.3 (1.3)	6.3 (1.3)	0.72 †
Initial random glucose, mg/dL (SD)	139.7 (59.4)	136.1 (54.3)	0.53 †
SBP, mmHg (SD)	147.6 (25.6)	142.3 (26.0)	0.91 †

Abbreviations: BMD, bone mineral density; SD, standard deviation; BMI, body mass index; NIHSS, National Institute Health of Stroke Scale; SVO, small vessel occlusion; LAA, large artery atherosclerosis; CE, cardioembolism; mRS, modified Rankin Scale; WBC, white blood cell; LDL, low density lipoprotein; HbA1c, glycated haemoglobin; SBP, systolic blood pressure. * Calculated by the chi-square test. † Calculated by Student's *t*-test

Table S2. Baseline characteristics according to femoral neck BMD (analyzed with database 1).

	Femoral neck BMD T score>-2.5 (*n* = 222)	Femoral neck BMD T score<-2.5 (*n* = 38)	*p*-value
Age, years (SD)	72.3 (10.7)	76.3 (8.8)	0.32†
Female (%)	121 (54.5)	32 (84.2)	0.001*
BMI, kg/m^2 (SD)	24.2 (3.9)	22.6 (4.0)	0.15†
Initial NIHSS score (%)			0.95*
0-4	154 (69.4)	26 (68.4)	
5~11	54 (24.3)	10 (26.3)	
>11	14 (6.3)	2 (5.3)	
Stroke subtype (%)			0.34*
SVO	62 (27.9)	11 (28.9)	
LAA	83 (37.4)	10 (26.3)	
CE	38 (17.1)	6 (15.8)	
Other	39 (17.6)	11 (28.9)	
Prior stroke (%)	51 (23.0)	15 (39.5)	0.04*
Hypertension (%)	141 (36.5)	29 (76.3)	0.14*
Diabetes mellitus (%)	81 (36.5)	11 (28.9)	0.46*

Hyperlipidaemia (%)	33 (14.9)	4 (10.5)	0.62*
Current smoking (%)	33 (14.9)	4 (10.5)	0.06*
Atrial fibrillation (%)	42 (18.9)	8 (21.1)	0.82*
Prior antithrombotic agent use (%)	93 (41.9)	18 (47.4)	0.60*
Education, years (SD)	6.9 (4.2)	5.8 (4.5)	0.51†
Lesions			0.18*
Supratentorial	156 (70.3)	91 (81.6)	
Infratentorial	66 (29.7)	7 (18.4)	
mRS>2 at discharge (%)	55 (24.8)	11 (28.9)	0.69*
mRS>2 at 3 months (%)	74 (33.3)	15 (39.5)	0.77*
WBC, ×1000/μL (SD)	7.5 (2.5)	7.3 (2.4)	0.81†
LDL, mg/dL (SD)	97.4 (33.8)	86.5 (26.5)	0.17†
Haemoglobin, g/dL (SD)	13.8 (1.8)	13.4 (1.9)	0.61†
Creatinine, mg/dL (SD)	0.97 (0.38)	1.20 (1.11)	0.002†
HbA1c, % (SD)	6.2 (1.3)	6.3 (1.5)	0.68†
Initial random glucose, mg/dL (SD)	148.1 (25.8)	142.7 (24.9)	0.53†
SBP, mmHg (SD)	148.4 (25.9)	144.2 (25.0)	0.57†

Abbreviations: BMD, bone mineral density; SD, standard deviation; BMI, body mass index; NIHSS, National Institute Health of Stroke Scale; SVO, small vessel occlusion; LAA, large artery atherosclerosis; CE, cardioembolism; mRS, modified Rankin Scale; WBC, white blood cell; LDL, low density lipoprotein; HbA1c, glycated haemoglobin; SBP, systolic blood pressure. * Calculated by the chi-square test. † Calculated by Student's *t*-test

Table S3. Baseline characteristics according to lumbar spine BMD (analyzed with database 1).

	Lumbar spine BMD T score>-2.5 (*n* = 209)	Lumbar spine BMD T score<-2.5 (*n* = 51)	*p*-value
Age, years (SD)	71.9 (10.9)	77.3 (7.5)	0.01†
Female (%)	105 (50.2)	48 (94.1)	<0.001*
BMI, kg/m^2 (SD)	24.3 (4.0)	22.5 (3.2)	0.36†
Initial NIHSS score (%)			0.23*
0-4	148 (70.8)	32 (62.7)	
5~11	47 (22.5)	17 (33.3)	
>11	14 (6.7)	2 (3.9)	
Stroke subtype (%)			0.52*
SVO	56 (26.8)	17 (33.3)	
LAA	78 (37.3)	15 (29.4)	
CE	37 (17.7)	7 (13.7)	
Other	38 (18.2)	12 (23.5)	
Prior stroke (%)	53 (25.4)	13 (25.5)	0.99*
Hypertension (%)	136 (65.1)	34 (66.7)	0.87*
Diabetes mellitus (%)	81 (38.8)	11 (21.6)	0.02*

Hyperlipidaemia (%)	31 (14.8)	6 (11.8)	0.66*
Current smoking (%)	34 (16.3)	0 (0.00)	0.002*
Atrial fibrillation (%)	43 (20.6)	7 (13.7)	0.33*
Prior antithrombotic agent use (%)	92 (44.0)	19 (37.3)	0.43*
Education, years (SD)	6.8 (4.3)	6.5 (4.3)	0.47†
Lesions			0.91*
Supratentorial	150 (71.8)	37 (72.5)	
Infratentorial	59 (28.2)	14 (27.5)	
mRS>2 at discharge (%)	52 (24.9)	14 (27.5)	0.72*
mRS>2 at 3 months (%)	70 (33.5)	19 (37.3)	0.62*
WBC, ×1000/μL (SD)	7.5 (2.6)	7.3 (2.2)	0.50†
LDL, mg/dL (SD)	97.6 (34.1)	88.7 (27.7)	0.10†
Haemoglobin, g/dL (SD)	1.8 (1.9)	13.3 (1.8)	0.86†
Creatinine, mg/dL (SD)	0.97 (0.37)	1.17 (1.01)	0.002†
HbA1c, % (SD)	6.3 (1.4)	6.2 (1.3)	0.42†
Initial random glucose, mg/dL (SD)	140.3 (62.4)	129.3 (43.2)	0.14†
SBP, mmHg (SD)	148.4 (25.7)	142.8 (25.2)	0.73†

Abbreviations: BMD, bone mineral density; SD, standard deviation; BMI, body mass index; NIHSS, National Institute Health of Stroke Scale; SVO, small vessel occlusion; LAA, large artery atherosclerosis; CE, cardioembolism; mRS, modified Rankin Scale; WBC, white blood cell; LDL, low density lipoprotein; HbA1c, glycated haemoglobin; SBP, systolic blood pressure. * Calculated by the chi-square test. † Calculated by Student's *t*-test

Table S4. Multivariable analysis showing the effects of several variables on cognitive impairment in acute phase (analyzed with database 1).

	Total BMD			
	Mild cognitive impairment vs no cognitive impairment		Severe cognitive impairment vs no cognitive impairment	
	OR (95% CI)	P-value	OR (95% CI)	P-value
Age	1.07 (1.03-1.12)	0.001	1.12 (1.06-1.17)	<0.001
Female sex	1.49 (0.68-3.28)	0.32	2.96 (1.23-7.15)	0.02
Years of education	0.89 (0.82-0.97)	0.01	0.76 (0.69-0.85)	<0.001
History of DM	1.47 (0.72-3.02)	0.29	0.84 (0.38-1.85)	0.66
Current smoking status	1.68 (0.57-4.97)	0.35	2.47 (0.70-8.76)	0.16
LDL level	1.01 (1.00-1.02)	0.04	1.01 (1.00-1.03)	0.04
Creatinine level	1.80 (0.72-4.50)	0.21	1.94 (0.76-4.94)	0.17
Fazekas scale	1.31 (0.83-2.06)	0.24	2.16 (1.32-3.52)	0.002
BMD T score<-2.5	2.64 (1.05-6.61)	0.04	2.22 (0.84-5.86)	0.11

	Femoral neck BMD			
	Mild cognitive impairment vs no cognitive impairment		Severe cognitive impairment vs no cognitive impairment	
	OR (95% CI)	P-value	OR (95% CI)	P-value
Age	1.08 (1.03-1.12)	<0.001	1.12 (1.07-1.18)	<0.001
Female sex	1.73 (0.81-3.68)	0.15	3.35 (1.41-7.99)	0.01
Years of education	0.89 (0.82-0.97)	0.01	0.76 (0.69-0.84)	<0.001
Prior stroke status	0.71 (0.29-1.71)	0.44	1.58 (0.64-3.90)	0.32
Current smoking status	1.51 (0.51-4.42)	0.46	2.80 (0.80-9.83)	0.11
LDL level	1.01 (1.00-1.02)	0.051	1.01 (1.00-1.03)	0.03

Creatinine level	1.71 (0.68-4.31)	0.26	1.89 (0.73-4.87)	0.19
Fazekas scale	1.34 (0.84-2.14)	0.22	1.96 (1.18-3.24)	0.01
Femoral BMD T score<-2.5	3.09 (0.87-10.99)	0.09	4.09 (1.11-15.14)	0.04

	Lumbar BMD			
	Mild cognitive impairment vs no cognitive impairment		Severe cognitive impairment vs no cognitive impairment	
	OR (95% CI)	P-value	OR (95% CI)	P-value
Age	1.07 (1.03-1.12)	<0.001	1.12 (1.06-1.17)	<0.001
Female sex	1.89 (0.86-4.15)	0.12	3.64 (1.51-8.77)	0.004
Years of education	0.89 (0.82-0.97)	0.01	0.76 (0.69-0.85)	<0.001
History of DM	1.32 (0.64-2.71)	0.45	0.76 (0.34-1.67)	0.49
Current smoking status	1.60 (0.54-4.72)	0.39	2.39 (0.67-8.46)	0.18
LDL level	1.01 (0.99-1.02)	0.08	1.01 (0.99-1.02)	0.06
Creatinine level	1.79 (0.74-4.37)	0.20	1.93 (0.77-4.80)	0.16
Fazekas scale	1.33 (0.84-2.08)	0.22	2.17 (1.33-3.55)	0.002
Lumbar BMD T score<-2.5	1.20 (0.44-3.24)	0.73	1.07 (0.38-3.02)	0.89

Table S5. Multivariable analysis showing the effects of several variables on cognitive impairment in recovery phase (analyzed with database 2).

	Total BMD			
	Mild cognitive impairment vs no cognitive impairment		Severe cognitive impairment vs no cognitive impairment	
	OR (95% CI)	P-value	OR (95% CI)	P-value
Age	1.02 (0.98-1.07)	0.25	1.13 (1.07-1.19)	<0.001
Female sex	1.29 (0.49-3.36)	0.61	1.33 (0.49-3.89)	0.58
Years of education	0.94 (0.85-1.04)	0.22	0.87 (0.78-0.97)	0.01
History of DM	1.04 (0.42-2.57)	0.93	1.21 (0.48-3.06)	0.69
Current smoking status	1.14 (0.35-3.71)	0.82	1.21 (0.33-4.48)	0.78
LDL level	1.00 (0.99-1.01)	0.94	1.00 (0.99-1.01)	0.95
Creatinine level	5.06 (0.80-31.89)	0.09	6.94 (1.09-44.24)	0.04
Fazekas scale	2.33 (1.27-4.28)	0.01	3.55 (1.92-6.56)	<0.001
BMD T score<-2.5	2.90 (0.80-10.52)	0.11	2.55 (0.69-9.51)	0.16

	Femoral neck BMD			
	Mild cognitive impairment vs no cognitive impairment		Severe cognitive impairment vs no cognitive impairment	
	OR (95% CI)	P-value	OR (95% CI)	P-value
Age	1.03 (0.99-1.07)	0.19	1.14 (1.08-1.20)	<0.001
Female sex	1.51 (0.59-3.83)	0.39	1.34 (0.51-3.53)	0.55
Years of education	0.94 (0.85-1.04)	0.23	0.97 (0.78-0.97)	0.01
Prior stroke status	0.70 (0.24-2.02)	0.5	0.80 (0.27-2.35)	0.68
Current smoking status	1.14 (0.35-3.71)	0.83	1.26 (0.34-4.70)	0.73

LDL level	0.999 (0.99-1.01)	0.89	0.999 (0.99-1.01)	0.88
Creatinine level	4.82 (0.76-31.78)	0.09	6.53 (0.99-43.03)	0.051
Fazekas scale	2.52 (1.34-4.73)	0.004	3.64 (1.92-6.89)	<0.001
Femoral BMD T score<-2.5	4.28 (0.43-42.21)	0.21	11.17 (1.12-110.98)	0.04

	Lumbar BMD			
	Mild cognitive impairment vs no cognitive impairment		Severe cognitive impairment vs no cognitive impairment	
	OR (95% CI)	P-value	OR (95% CI)	P-value
Age	1.02 (0.98-1.06)	0.31	1.14 (1.08-1.20)	<0.001
Female sex	1.32 (0.50-3.44)	0.57	1.57 (0.59-4.22)	0.37
Years of education	0.94 (0.85-1.04)	0.23	0.88 (0.79-0.97)	0.01
History of DM	1.13 (0.45-2.81)	0.8	1.18 (0.47-2.99)	0.73
Current smoking status	1.11 (0.35-3.59)	0.8600	1.14 (0.31-4.21)	0.85
LDL level	1.00 (0.99-1.01)	0.99	0.999 (0.99-1.01)	0.83
Creatinine level	5.01 (0.78-32.09)	0.09	7.00 (1.08-45.32)	0.04
Fazekas scale	2.39 (1.30-4.40)	0.01	3.57 (1.93-6.62)	<0.001
Lumbar BMD T score<-2.5	2.94 (0.68-12.61)	0.15	1.53 (0.35-6.70)	0.60

Table S6. Multivariable analysis showing the effects of several variables on WMD (analyzed with database 3).

	Total BMD			
	Moderate WMD vs normal to mild WMD		Severe WMD vs normal to mild WMD	
	OR (95% CI)	P-value	OR (95% CI)	P-value
Age	1.05 (1.02-1.08)	0.003	1.08 (1.04-1.13)	<0.001
Female sex	0.74 (0.38-1.43)	0.37	1.07 (0.53-2.18)	0.85
Years of education	0.95 (0.88-1.01)	0.11	0.91 (0.85-0.98)	0.02
History of DM	0.98 (0.54-1.77)	0.94	0.91 (0.48-1.72)	0.78
Current smoking status	0.93 (0.36-2.37)	0.88	1.44 (0.53-3.90)	0.48
LDL level	0.99 (0.99-1.003)	0.18	0.997 (0.998-1.01)	0.51
Creatinine level	1.81 (0.81-4.06)	0.15	1.56 (0.67-3.63)	0.31
BMD T score<-2.5	1.95 (0.97-3.91)	0.06	1.05 (0.49-2.24)	0.91

	Femoral neck BMD			
	Moderate WMD vs normal to mild WMD		Severe WMD vs normal to mild WMD	
	OR (95% CI)	P-value	OR (95% CI)	P-value
Age	1.05 (1.02-1.09)	0.002	1.08 (1.04-1.12)	<0.0001
Female sex	0.83 (0.44-1.56)	0.56	0.999 (0.48-2.05)	0.997
Years of education	0.94 (0.88-1.01)	0.10	0.90 (0.84-0.97)	0.01
Prior stroke status	1.32 (0.64-2.70)	0.46	3.57 (1.74-7.34)	0.001
Current smoking status	0.93 (0.37-2.34)	0.87	1.57 (0.57-4.33)	0.39

LDL level	0.99 (0.99-1.003)	0.22	0.998 (0.99-1.01)	0.68
Creatinine level	1.86 (0.80-4.31)	0.15	1.65 (0.68-3.999)	0.27
Femoral BMD T score<-2.5	2.62 (0.99-6.90)	0.052	3.06 (1.14-8.38)	0.03

	Lumbar BMD			
	Moderate WMD vs normal to mild WMD		Severe WMD vs normal to mild WMD	
	OR (95% CI)	P-value	OR (95% CI)	P-value
Age	1.05 (1.02-1.09)	0.002	1.09 (1.05-1.13)	<0.001
Female sex	0.86 (0.45-1.65)	0.65	1.16 (0.58-2.34)	0.68
Years of education	0.95 (0.88-1.01)	0.11	0.92 (0.85-0.99)	0.02
History of DM	0.94 (0.52-1.69)	0.82	0.88 (0.47-1.67)	0.70
Current smoking status	0.90 (0.35-2.28)	0.82	1.41 (0.52-3.84)	0.50
LDL level	0.99 (0.99-1.002)	0.15	0.996 (0.99-1.01)	0.44
Creatinine level	1.80 (0.81-4.00)	0.15	1.58 (0.68-3.66)	0.29
Lumbar BMD T score<-2.5	1.27 (0.61-2.65)	0.53	0.81 (0.31-1.63)	0.42

Table S7. Interaction analysis showing whether WMD modified the association between BMD and cognitive impairment.

	P-value for the interaction in the acute phase		P-value for the interaction in the recovery phase	
	Moderate cognitive impairment	Severe cognitive impairment	Moderate cognitive impairment	Severe cognitive impairment
Total BMD	0.42	0.19	<0.001	<0.001
Femoral neck BMD	0.84	0.93	<0.001	<0.001
Lumbar spine BMD	0.83	0.20	<0.001	<0.001

Abbreviations: WMD, white matter disease; BMD, bone mineral density; OR, odds ratio; CI, confidence interval

Table S8. Multivariable analysis: association between BMD and categorized MoCA scales (analyzed using databases 1 and 2).

	MoCA score of 11~17 vs MoCA score of 18~30		MoCA score of 0~10 vs MoCA score of 18~30	
	Adjusted OR (95% CI) in the acute phase	Adjusted OR (95% CI) in the recovery phase	Adjusted OR (95% CI) in the acute phase	Adjusted OR (95% CI) in the recovery phase
Total BMD T score<-2.5[a]	3.09 (1.25-7.63)	2.05 (0.65-6.47)	2.23 (0.88-5.67)	1.75 (0.55-5.51)
Femoral neck BMD T score<-2.5[b]	2.57 (0.79-8.38)	5.10 (0.53-48.99)	3.44 (1.03-11.51)	12.49 (1.30-119.96)
Lumbar spine BMD T score<-2.5[a]	1.78 (0.51-6.19)	1.78 (0.51-6.19)	1.30 (0.48-3.56)	0.95 (0.28-3.30)

Abbreviations: BMD, bone mineral density; OR, odds ratio; CI, confidence interval

[a]Adjusted for age, female sex, years of education, diabetes mellitus status, current smoking status, low-density lipoprotein level, creatinine level and the Fazekas score

[b]Adjusted for age, female sex, years of education, prior stroke status, current smoking status, low-density lipoprotein level, creatinine level and the Fazekas score

Table S9. Linear regression analysis: volumetric BMD and K-MMSE score.

	B	SE B	β	p	R	R^2	Adjusted R^2
Femoral neck BMD T score <-2.5 (acute phase)[1]	9.606	2.696	0.226	<0.001	0.526	0.277	0.260
Femoral neck BMD T score <-2.5 (recovery phase)[1]	8.575	3.071	0.186	0.01	0.602	0.363	0.338
Lumbar spine BMD T score <-2.5 (acute phase)[2]	2.974	2.783	0.072	0.286	0.477	0.227	0.209
Lumbar spine BMD T score <-2.5 (recovery phase)[2]	4.960	2.924	0.113	0.091	0.590	0.348	0.323

Abbreviations: BMD, bone mineral density; K-MMSE, Korean mini-mental status examination. [1]adjusted for age, female sex, years of education, prior stroke status, current smoking status, low density lipoprotein, creatinine level and Fazekas's scale. [2]adjusted for age, female sex, years of education, diabetes mellitus status, current smoking status, low density lipoprotein, creatinine level and Fazekas's scale

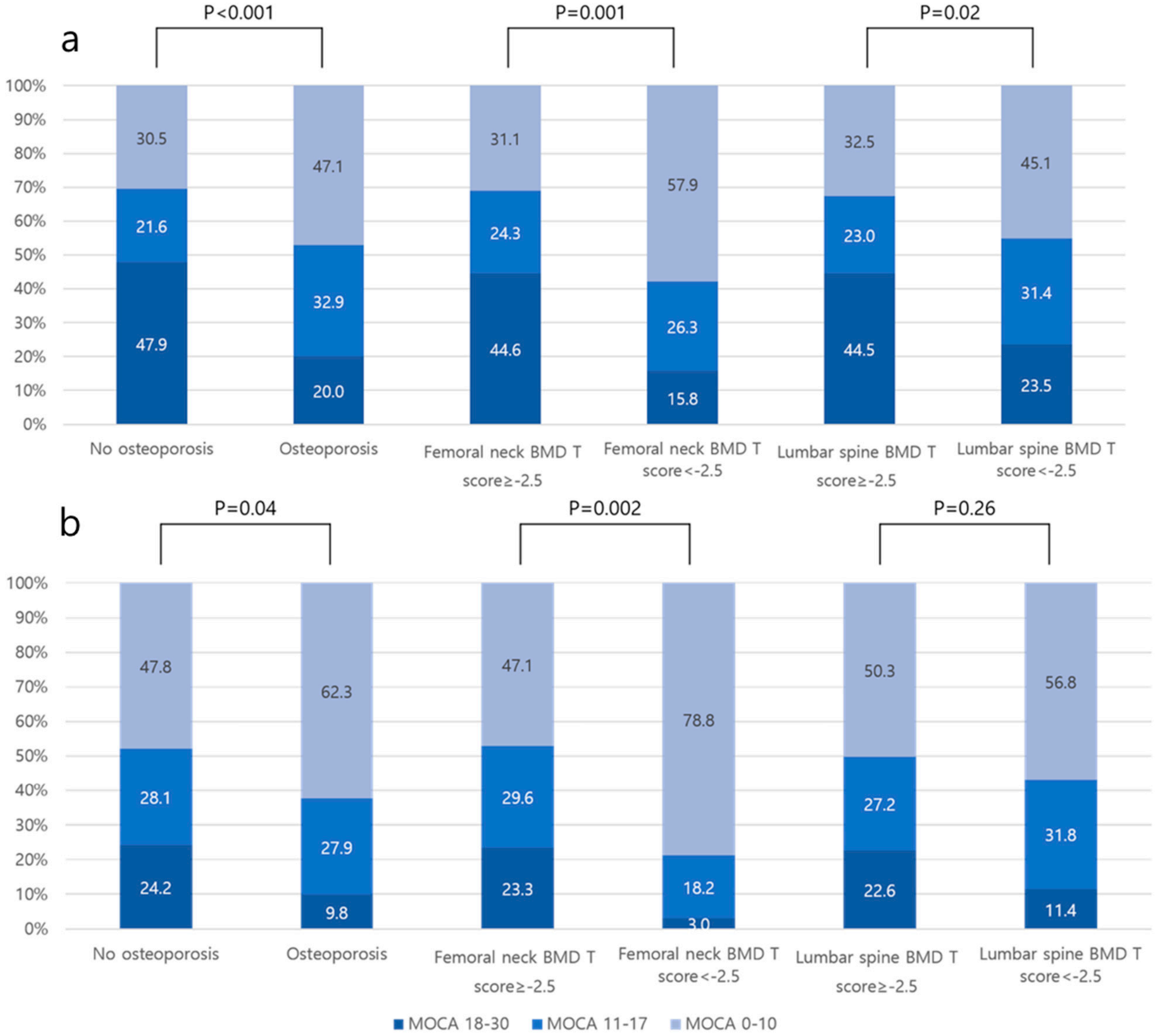

Figure. S1. Distributions of categorized MoCA scores according to BMD (total BMD, femoral neck BMD and lumbar spine BMD) during the acute phase (**a**) and recovery phase of ischemic stroke (**b**).
